# Mapping the adaptive landscape of a major agricultural pathogen reveals evolutionary constraints across heterogeneous environments

**DOI:** 10.1038/s41396-020-00859-w

**Published:** 2021-01-15

**Authors:** Anik Dutta, Fanny E. Hartmann, Carolina Sardinha Francisco, Bruce A. McDonald, Daniel Croll

**Affiliations:** 1 Plant Pathology, Institute of Integrative Biology, ETH Zurich, Zurich, Switzerland; 2 Ecologie Systématique Evolution, CNRS, Université Paris-Saclay, AgroParisTech, 91400 Orsay, France; 3 Laboratory of Evolutionary Genetics, Institute of Biology, University of Neuchâtel, 2000 Neuchâtel, Switzerland; 4 Present Address: Environmental Genomics Group, Botanical Institute, CAU Kiel, Germany

**Keywords:** Population genetics, Plant sciences, Molecular evolution, Fungi

## Abstract

The adaptive potential of pathogens in novel or heterogeneous environments underpins the risk of disease epidemics. Antagonistic pleiotropy or differential resource allocation among life-history traits can constrain pathogen adaptation. However, we lack understanding of how the genetic architecture of individual traits can generate trade-offs. Here, we report a large-scale study based on 145 global strains of the fungal wheat pathogen *Zymoseptoria tritici* from four continents. We measured 50 life-history traits, including virulence and reproduction on 12 different wheat hosts and growth responses to several abiotic stressors. To elucidate the genetic basis of adaptation, we used genome-wide association mapping coupled with genetic correlation analyses. We show that most traits are governed by polygenic architectures and are highly heritable suggesting that adaptation proceeds mainly through allele frequency shifts at many loci. We identified negative genetic correlations among traits related to host colonization and survival in stressful environments. Such genetic constraints indicate that pleiotropic effects could limit the pathogen’s ability to cause host damage. In contrast, adaptation to abiotic stress factors was likely facilitated by synergistic pleiotropy. Our study illustrates how comprehensive mapping of life-history trait architectures across diverse environments allows to predict evolutionary trajectories of pathogens confronted with environmental perturbations.

## Introduction

Adaptation to heterogeneous environments (both biotic and abiotic) is critically important for pathogens to successfully infect and colonize their hosts, disseminate to new hosts and cause epidemics, and survive in the absence of their host. Adaptation to new environments is contingent on genetic variation in life-history traits. As a consequence, adaptation can be severely constrained in pathogen populations of low genetic diversity. Furthermore, trade-offs between advantageous traits can impose limits on adaptive evolution [1, 2]. Trade-offs typically arise from differential resource allocation and antagonistic gene actions, including pleiotropy, that inhibit the simultaneous increase in two favorable traits [3–5]. Understanding genetic routes of adaptation helps to predict evolutionary responses to changing environmental conditions [6, 7]. Despite the likely importance of trade-offs, we largely lack evidence regarding how pathogen adaptation in natural environments was shaped by potential constraints. A few studies focusing on specific traits and growth conditions reported mutations conferring thermal trade-offs in *Escherichia coli* [8], fitness trade-offs in drug resistance in *Candida albicans* [9], and a simultaneous increase in melanin synthesis and virulence in *Aspergillus fumigatus* [10]. The mutations underlying synergistic or antagonistic interactions among traits, including virulence and environmental stress adaptation, remain largely unknown, hindering our understanding of adaptive landscapes in pathogens.

Trade-offs are defined as phenotypic correlations that prevent organisms from reaching maximum fitness in specific environments and are often influenced by environmental conditions. Trade-offs impose evolutionary constraints if the expression of each trait is governed by the same mutations [4]. Pleiotropy occurs when single mutations simultaneously affect the expression of several traits, generating genetic correlations among these traits [11, 12]. Genetic correlations can also arise from linkage disequilibrium among mutations, but such correlations are less stable over generations as recombination tends to break down the association [6, 13, 14]. Investigation of pleiotropic effects has largely focused on a small number of traits in different species [15–19]. To elucidate the constraints on adaptation in pathogens will require a genome-wide perspective that can consider trade-offs and pleiotropy across a broad spectrum of fitness-relevant life-history traits. It is particularly challenging to identify trade-offs and pleiotropy when the genetic architecture of the studied traits is dominated by numerous small effect loci (i.e., polygenic traits). Importantly, the majority of phenotypes affecting fitness show such polygenic genetic architectures [20]. We aimed to establish a fine-scale map of genetic correlations to unravel constraints on trait evolution in host and non-host environments.

*Zymoseptoria tritici* is a major fungal pathogen that poses a significant threat to global wheat production by causing septoria tritici blotch (STB) disease [21, 22]. The life cycle of the pathogen is complex, including niche adaptations that enable growth and survival on host tissue across a wide range of temperatures, asexual reproduction that enables short distance dispersal and the production of survival propagules at the end of the growing season [23]. The pathogen causes necrotic lesions on infected leaves and reproduces asexually with fruiting bodies called pycnidia that form within lesions and produce spores that are dispersed by rain splash. Lesion development and pycnidia production show strong correlations at the phenotypic level consistent with trade-offs [24]. Hence, *Z. tritici* virulence (i.e., damage through lesions) and reproduction (i.e., spore production in pycnidia) may be constrained at the genetic level. Later in the infection cycle, the pathogen forms sexual ascospores that are dispersed over long distances by wind. Across the global distribution range, populations vary in their degree of thermal adaptation and fungicide resistance as a result of differences in temperature and fungicide exposure [25, 26]. The pathogen produces variable levels of melanin under stress conditions and melanin production shows negative correlations with growth rates and fungicide susceptibility [17, 27]. Under high temperature, the pathogen also produces resistant survival structures called chlamydospores [28]. As for most pathogens, the genetic architecture underlying life history traits, including virulence, reproduction and stress resistance remains largely unknown. The *Z. tritici* model offers powerful experimental approaches to map phenotypic trait architectures using genome-wide association mapping (GWAS; [29]), shows high heritable trait variation in populations [30] and extensive genomic resources are available [31, 32] that can enable the precise localization of adaptive loci.

Here, we perform large-scale GWAS in a global collection of 145 *Z. tritici* isolates originating from five field populations spanning four continents. We map adaptive loci for a total of 50 phenotypic traits, including virulence and reproduction on 12 distinct host genotypes covering both landrace diversity and elite cultivars. Adaptation to environmental factors was assessed for different temperatures and fungicide concentrations. Combining information about adaptive loci across the genome, we analyze the extent of genetic correlations among classes of phenotypic traits to identify major trade-offs. By mapping the adaptive landscape of constraints and facilitation governing phenotypic trait evolution, we generate a comprehensive view on the genetic underpinnings of pathogen evolution.

## Materials and methods

### Fungal material

We used a panel of 145 *Z. tritici* isolates (Supplementary Table S1) sampled separately from four wheat fields. Three fields were planted with a single wheat cultivar: in Australia (*n* = *27*), Israel (*n* = *30*), two nearby field sites in Switzerland (*n* = *2* and *n* = *30*). The field population in the USA was sampled from two distinct cultivars growing in a randomized block design: Oregon.R, *n* = *26*; Oregon.S, *n* = *30* All samplings were made between 1990 and 2001 ([33]; Supplementary Table S1). The populations Oregon.R and Oregon.S were sampled from the moderately resistant wheat cultivar Madsen and the susceptible cultivar Stephens, respectively. Field populations were screened for clones and the analyzed set contains only genetically distinct isolates [33, 34]. Since collection, spores of each isolate were stored and maintained in 50% glycerol or anhydrous silica at −80 °C.

### Whole-genome sequencing and single nucleotide polymorphism calling

In addition to the already deposited Illumina whole genome sequencing data for 130 isolates [30], we generated raw sequence data for 15 additional isolates (see Supplementary Table S1 for all accession numbers). Briefly, high-quality genomic DNA from each isolate was extracted following the DNeasy Plant Mini Kits (Qiagen) protocol and paired-end sequencing of 100 bp with an ~500 bp insert size was performed using the Illumina HiSeq2000 platform. The newly generated raw sequences were deposited under the BioProject PRJNA327615. We used Trimmomatic v.0.36 [35] to trim low-quality sequencing reads and remove adapter contamination in each isolate. The refined sequences were aligned to the *Z. tritici* reference genome IPO323 [32] using Bowtie2 v.2.3.3 [36] and duplicate sequences were removed using MarkDuplicates in Picard tools v.1.118 (http://broadinstitute.github.io/picard). We used Genome Analysis Toolkit (GATK) v.4.0.1.2 [37] for single nucleotide polymorphism (SNP) calling and variant filtration. The GATK HaplotypeCaller was used on each isolate with the command -emitRefConfidence GVCF and -sample_ploidy 1 (*Z. tritici* is haploid). Joint variant calls were performed using GenotypeGVCFs on a merged gvcf variant file with the option -maxAltAlleles 2. We used VariantFiltration and SNPs were removed if any of the following filter conditions applied: QUAL < 250 (overall quality filter); QD < 20.0 (avoiding quality inflation in high-coverage regions); MQ < 30.0 (avoid calls from ambiguously mapped reads); −2 > BaseQRankSum > 2; −2 > MQRankSum > 2; −2 > ReadPosRankSum > 2; FS > 0.1. The genotyping accuracy was previously investigated and found to be highly congruent with alternative SNP callers [38]. The final dataset was obtained by filtering with a genotyping call rate of ≥80% and minor allele frequency (MAF) > 5%, which resulted in 716'619 biallelic SNPs across all 21 chromosomes.

### In planta phenotyping

We used phenotypic data (Supplementary Table S2) on pathogen virulence and reproduction obtained by Dutta et al. [24] on a panel of 12 genetically different wheat populations including five landraces (Chinese Spring, 1011, 1204, 4391, and 5254), six commercial varieties (Drifter, Gene, Greina, Runal, Titlis, Toronit) and a back-cross line (Arina*Lr34*). Arina*Lr34* carries the wheat leaf rust resistance gene *Lr34*, which provides resistance to wheat stripe rust [39] and powdery mildew [40] but was not previously tested for resistance to *Z. tritici*. The 1011, 1204, 4391, and 5254 landraces were selected from the Swiss National Gene Bank (www.bdn.ch). Six pots with three seeds of each cultivar were sown per pot and placed on a tray in a 2 × 3 array. The experiment was divided into two phases (6 cultivars at a time) due to space limitations. The trays were kept at 22 °C (day) and 18 °C (night) with 70% relative humidity (RH) and a 16-h photoperiod in a greenhouse chamber. Each tray containing two-week-old seedlings of six cultivars was inoculated uniformly with each isolate using an airbrush spray gun until run-off. Three independent inoculations were performed to generate three biological replications in different greenhouse chambers in both phases. All leaves from each host-by-isolate combination were collected on the same day between 19–26 days post inoculation (dpi) and analyzed using automated image analysis (AIA; [41]). The AIA provided quantitative estimates of the necrotic lesion area and pycnidia density within the lesion area. The lesion area and pycnidia density were used as proxies for virulence and reproduction, respectively. The phenotyping procedures are described in more detail in Dutta et al. [24].

### In vitro phenotyping

Fungal colony growth rate (mm per day), temperature sensitivity, mean colony area, fungicide resistance, and melanization in the presence or absence of fungicide were measured in vitro (Supplementary Table S3). Data on the morphological stress response (i.e., formation of chlamydospores or hyphae) was obtained from Francisco et al. [42]. The methods used for the in vitro phenotyping were adopted from earlier studies [17, 43–45]. Briefly, each isolate was regenerated from long-term storage conditions and grown on Petri dishes containing yeast malt sucrose agar (4 gl^−1^ yeast extract, 4 gl^− 1^ malt extract, 4 gl^−1^ sucrose, 50 mgl^−1^ kanamycin) for four to five days at 18 °C. Blastospore suspensions were collected from each plate by adding 0.6 ml of sterile water and diluted to a final concentration of 200 spores/ml using KOVA counting slides (Hycor Biomedical, Inc., Garden Grove, CA, USA). For each isolate, a 500 µl spore suspension was spread on Petri plates containing potato dextrose agar (PDA, 4 gl^−1^ potato starch, 20 gl^−1^ dextrose, 15 gl^−1^ agar) using a sterile glass rod. Plates were kept at 15 and 22 °C at 70% RH. Each plate was photographed at 8, 11 and 14 dpi with a digital camera.

The digital images of each plate from five technical replications were used for AIA in ImageJ following the scripts by Lendenmann et al. [44] to obtain colony area information for each isolate. Estimates are based on an average of 45 spore colonies. The mean colony radii taking the square-root of mean colony area (*√(mean colony area/π)*) were estimated and fitted over the three time points (8, 11, and 14 dpi) following a general linear model to obtain growth rates (mm/day) at 15 and 22 °C. Temperature and fungicide sensitivity of each isolate was calculated using the growth rate ratio between 15 and 22 °C, between 22 °C and growth rate at 22 °C on PDA amended with propiconazole (Syngenta, Basel, Switzerland; 0.05 ppm), respectively. In addition, we used mean colony area measured for each isolate at 14 dpi on PDA at 15 °C (MCA_15 °C_14_dpi), 22 °C (MCA_22 °C_14_dpi), and 22 °C amended with 0.05 ppm propiconazole (MCA_14_dpi_azole), to estimate the ratio of colony area in cold (RCA_14_dpi) and fungicide environments (RCA_14_dpi_azole) compared to the control environment.

Fungicide resistance assays were carried out using propiconazole on microtiter plates. Growth inhibition was tested by growing spores adjusted to a spore concentration of 2.5 × 10^4^ spores/ml) in Sabouraud-dextrose liquid medium with the following concentrations of propiconazole: 0.00006, 0.00017, 0.0051, 0.0086, 0.015, 0.025, 0.042, 0.072, 0.20, 0.55, 1.5 mg/L and a control without fungicide. Each microtiter well was filled with 100 μl of medium at a given concentration of fungicide and 100 μl of spore suspension using the above spore concentration. Three technical replicates were used for each isolate. The plates were sealed and incubated in the dark for four days at 22 °C with 80% relative humidity. Fungal growth was measured with an Elisa plate reader (MR5000, Dynatech) by examining the optical density (OD) at 605 nm wave length. The growth inhibition at different fungicide concentrations was used to estimate the dose-response curves. The dose-response curves were used to estimate the EC_50_ value for each isolate using the drc v.3.0-1package [46] in the R-studio [47].

We estimated melanization of each isolate at 8, 11, 14, and 18 dpi grown in three different conditions, (i) 15 °C, (ii) 22 °C, (iii) 22 °C in the presence of fungicide (0.05 ppm propiconazole). The same protocol described for growth assays above was also used to assess fungicide resistance. At each time point, digital images of each plate were captured and analyzed using the AIA protocol described in Lendenmann et al. [44]. We recorded measures of gray value ranging from 0 (black) to 255 (white). By using the gray value measurement from each colony on each plate and replication, we obtained the mean gray value estimate for each isolate. For a more intuitive metric of melanization, we subtracted each mean gray value from 255 to obtain a scale for melanization ranging from 0 (white) to 255 (black).

### Statistical analyses

We used log-transformed least-square means (LSmeans) for each host×isolate combination and for each *in planta* trait obtained by Dutta et al. [24]. The host specialization (i.e., the affinity of isolates for specific hosts to maximize trait performance) index for each isolate was represented by the adjusted coefficient of variation (i.e., logarithm of adjusted variance/mean) of LSmeans for reproduction among all 12 hosts [24, 48]. The LSmeans of each isolate for all the in vitro traits was extracted using a one-way analysis of variance. To visualize trait variation among the isolates, we constructed a clustered heatmap with the R package ComplexHeatmap v.2.2.0 [49] using the *z-scores* (mean = 0 and sd = 1) of LSmeans for each isolate. The *z-scores* were used to reduce variability among different traits as they were expressed in different units. We excluded the morphological stress response data from the heatmap as clustering did not allow for binary data. The hierarchical clustering was performed following the agglomerative algorithm “complete” based on Euclidean distances.

We performed a genome wide association study (GWAS) for all 50 traits and 145 isolates based on 716'619 SNPs. Prior to GWAS, we conducted a principal component analysis (PCA) on the SNP dataset to investigate the population structure using TASSEL v.20200220 [50]. We constructed a genetic relatedness matrix (GRM) among isolates using all the genome-wide SNPs following the normalized identity-by-descent method [51] implemented in TASSEL v.20200220 [50]. Both PCA and GRM can be used to control the inflation of false positive SNPs due to population structure and individual relatedness in a GWAS. However, principal components were excluded from the GWAS as the Bayesian information criterion (BIC; [52]; Supplementary Table S4) test in R package GAPIT v.3.0 [53] indicated that the inclusion of principal components as covariate did not meaningfully improve the model. Thus, all GWAS were performed using a mixed linear model (MLM + K; [54]) where K is the previously estimated GRM used as a random factor in GAPIT. Considering the relatively small sample size (*n* = 145), we used an MLM instead of a compressed MLM, which groups individuals according to phenotypes following the implementation in GAPIT [55]. The following mixed linear model was used: *y* = *Xb* +*Zu* + *e*, where *y* is the vector of phenotypes, *X* and *Z* are the known design matrices, *b* is the unknown vector of fixed effects (SNPs), *u* is the vector of random effects (based on the GRM) and *e* is the vector of residual effects.

We used the stringent Bonferroni threshold (α = 0.05), false discovery rate (FDR) at 5 and 10% to retrieve SNPs at different levels of statistical confidence using the R package q-value [56]. For the FDR, we followed the Benjamini and Hochberg [57] method to identify significant genome-wide SNPs. We used the physical position of all SNPs above FDR 10% as coordinates to extract the closest genes following the latest annotation of the reference genome IPO323 [32, 58]. For this, we used the “closest” command in BEDtools version 2.29.0 [59]. Encoded proteins were assigned to protein families (PFAM) and gene ontology (GO) terms using InterProScan version 5.36–75.0 with default parameters [60]. Protein secretion signals were predicted using SignalP version 4.01 [61], Phobius [62] and TMHMM version 2.0 [63]. GO enrichment analyses were performed using the packages GSEABase version 1.35.0 and GOstats version 2.38.1 in R [64]. We used the false discovery rate of 0.01 as a cut-off and considered only GOs with a minimum term size of five genes in the genome for hypergeometric tests.

The SNP-based heritability (*h*^*2*^_*snp*_; equivalent to narrow-sense heritability) for each trait was estimated using the genome-wide complex trait analysis (GCTA) tool v.1.93.0 [51]. The *h*^*2*^_*snp*_ was estimated using a genome-based restricted maximum likelihood (GREML) approach using the phenotypic data of each trait and considering the additive effect of all the SNPs represented by the GRM. The tool uses the GRM as a random factor and estimates the proportion of phenotypic variance attributed to the random factor. GCTA estimates the genetic relatedness between two individuals (following the same approach as implemented in TASSEL, see above) from all the genome-wide SNPs following the formula from Yang et al. [51]:

$$A = \frac{1}{N}\,\sum_{i = 1}^N {\frac{{\left( {x_{ij} - 2p_i} \right)\left( {x_{ik} - 2p_i} \right)}}{{2p_i\left( {1 - p_i} \right)}}}$$

Where *x*_*i*j_ is the number of copies of the reference allele for the *i*th SNP of the *j*th individual and *p*_*i*_ is the frequency of the reference allele and *N* is the number of SNPs. As *Z. tritici* is haploid, the average relatedness among all pairs of unrelated individuals (off-diagonals of the GRM) was close to two as opposed to the expected value of one for humans [65]. The resulting GRM was used as a random effect in the mixed model to predict the phenotypic similarity among the individuals. It is important to note that the GRM was constructed based on all SNPs irrespective of whether a SNP was significantly associated to phenotypic trait variation or not. Thus, the heritability estimates based on the GRM are as accurate as the GRM, which is an approximation to the genetic similarities at causal SNPs [66]. Hence, heritability estimates based on the GRM are expected to be most accurate for polygenic traits (i.e., showing associations with numerous SNPs of weak effects).

To investigate whether traits are under shared genetic control, we estimated genetic correlations between all possible trait pairs following a bivariate GREML approach in GCTA [51, 67] including individual phenotypic trait values and the GRM. The correlation between the additive genetic values of two traits is regarded as the genetic correlation (*r*_*g*_). The bivariate GREML approach estimates the genetic covariance (*σ*_g12_) by quantifying whether two individuals tend to show phenotypic similarity considering their genetic similarity (based on the GRM). Here, the variance-covariance structure of the genetic values is described by the GRM and the phenotype is modeled as a function of individual genetic values. Genetic correlation coefficients are estimated as the ratio of the genetic covariance (*σ*_g12_) and genetic variance of two traits (*σ*_g1_ and *σ*_g2_). We visualized genetic correlation coefficients as a network using the R package “qgraph” [68]. Phenotypic correlations among all traits were analyzed with the *z-*scores of the phenotypes based on Pearson’s correlation coefficients. Multiple testing adjustments of *p* values were performed by following the Benjamini and Hochberg [57] method at *α* = 0.05. As the morphological stress response was recorded as a binary trait, we used a point biserial correlation [69] to correlate the trait with other quantitative phenotypes using the R package ltm v.1.1-1 [70].

## Results

### Extensive trait variability in a highly polymorphic pathogen

**Fig. 1:**
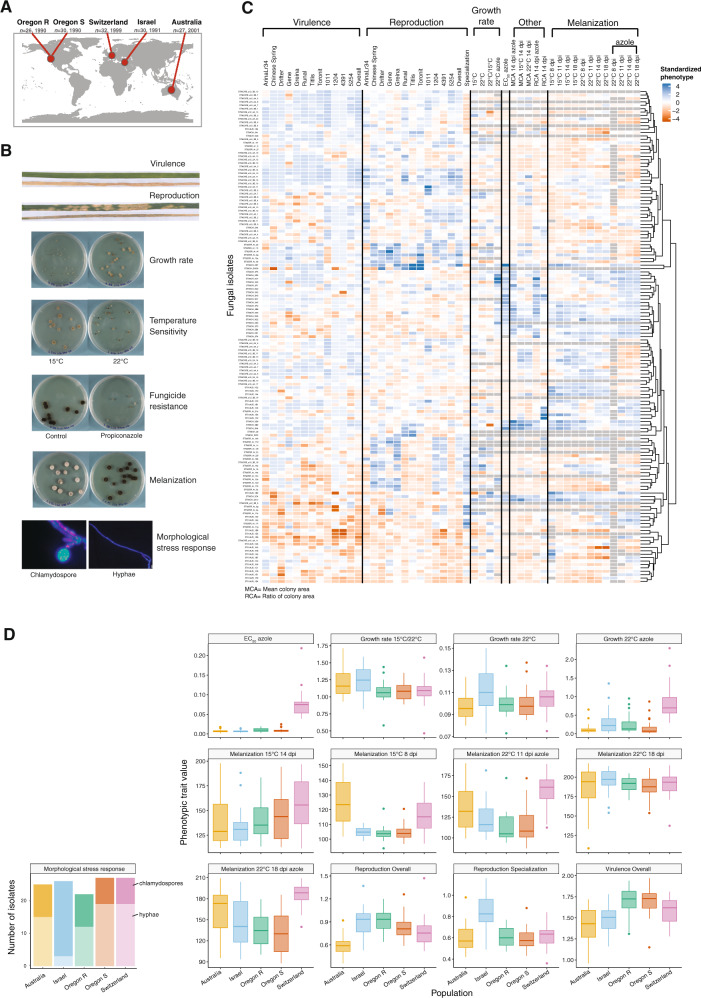
Geographic origin of populations and phenotypic diversity of *Zymoseptoria tritici*. **A** World map showing the field locations of the five pathogen populations (*n* = 145 isolates). The two populations in Oregon (USA) were sampled from wheat cultivars Madsen (resistant; Oregon.R) and Stephens (susceptible; Oregon.S). **B** Major categories of life-history traits observed in *Z. tritici* on the host and in the non-host environments (virulence – amount of necrotic lesion on the leaf; reproduction – pycnidia formation within lesions). **C** Heatmap showing phenotypic diversity of 49 traits using standardized phenotypic trait values. Pathogen virulence was assessed by the percentage of necrotic lesions on wheat leaves. Reproduction specialization was defined by the adjusted coefficient of variation of means across all 12 wheat hosts. Melanization was expressed on a grayscale ranging from 0 (white) to 255 (black). Dendrogram branches correspond to Euclidean distances for phenotypic trait values. **D** Phenotypic trait distribution in different environments among the five populations. The binary morphological stress response trait is shown separately.

We analyzed heritable phenotypic trait variation among *Z. tritici* isolates collected across the distribution range of the pathogen including the Middle East (the center of origin), Europe, North America and Australia (Fig. 1A). We quantified the expression of life-history and stress-response traits by exposing the individual isolates to different environmental conditions. To assess variation in pathogenicity traits, we infected 12 distinct wheat cultivars and measured the capacity to cause damage and reproduce on the host. We also assessed growth under culture conditions applying a variety of stress factors including temperature variation and exposure to fungicides (Fig. 1B). Isolates showed a high degree of phenotypic diversity across all measured traits (Fig. 1C & Supplementary Table S5). Trait expression was largely quantitative and almost all traits showed substantial variation within each field population. While there was no obvious geographic structure in phenotypic trait variation, we found considerable differences in mean trait values among populations (Fig. 1D). The population from Israel showed the highest degree of host specialization for reproduction and also the highest percentage of isolates that formed resistance structures (i.e., chlamydospore) under stress [42]. The Swiss population showed the highest fungicide resistance consistent with the earlier and more pervasive application of azole fungicides in Europe compared to other regions [25]. The extensive variation in phenotypic traits across host and non-host environments suggests that these traits are governed by complex genetic architectures. Hence, identifying the loci underpinning the trait variation across genotypes can provide insights into the evolutionary trajectory of these pathogen populations.

### Genetic architecture of host adaptation and survival traits

**Fig. 2:**
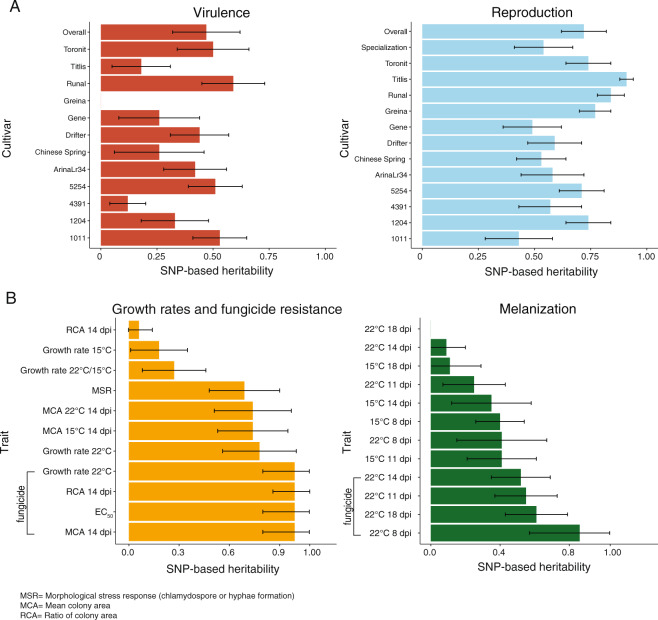
Phenotypic variance explained by common genetic variants. **A** Pathogen virulence (amount of necrotic lesion area) and reproduction (pycnidia density within the lesion area) were measured on 12 wheat hosts. **B** Growth rate in absence of the host, fungicide resistance, melanization and morphological stress response (chlamydospore or mycelium production). SNP-based heritabilities (*h*^*2*^_*snp*_) were estimated by following a genomic based restricted maximum likelihood GREML approach. Error bars indicate standard errors.

We estimated heritability (*h*^*2*^_*snp*_) for each of the traits using a genome-based restricted maximum likelihood (GREML) approach. To account for genetic relatedness among isolates, we used genome-wide SNP data as a random effect. The estimated *h*^*2*^_*snp*_ for virulence ranged from 0 to 0.59 (standard error, SE = 0.14) and for reproduction from 0.43 (SE = 0.15) to 0.91 (SE = 0.03; Fig. 2A). We found a higher mean *h*^*2*^_*snp*_ for reproduction (0.72, SE = 0.10) compared to the mean *h*^*2*^_*snp*_ for virulence (0.47, SE = 0.15). The higher heritability suggests that the short-term response to selection may be faster for reproduction. The heritability for growth traits, fungicide resistance, morphological stress response and thermal tolerance traits ranged widely from 0 to 0.99 with fungicide resistance traits showing the highest heritability (Fig. 2B). High heritability of traits assessed under culture conditions is expected due to the highly controlled environment. Compared to this, trait heritability assessed in greenhouse infection experiments is expected to be overall lower because of greater challenges associated with performing infection assays on plants. However, heritability comparisons among infection traits and cultivars, as well as among colony growth experiments (i.e., temperature variation and melanization) provide meaningful contrasts. The genetic basis of most assessed traits remains poorly unknown. Infection traits can be governed by a smaller number of major effect loci or show largely quantitative distributions [30]. Resistance to azole fungicides is thought to primarily mediated by mutations in the gene encoding the target of azoles (i.e., *CYP51*; [71]). However, GWAS for azole resistance identified a multitude of minor effect loci [72, 73].

**Fig. 3:**
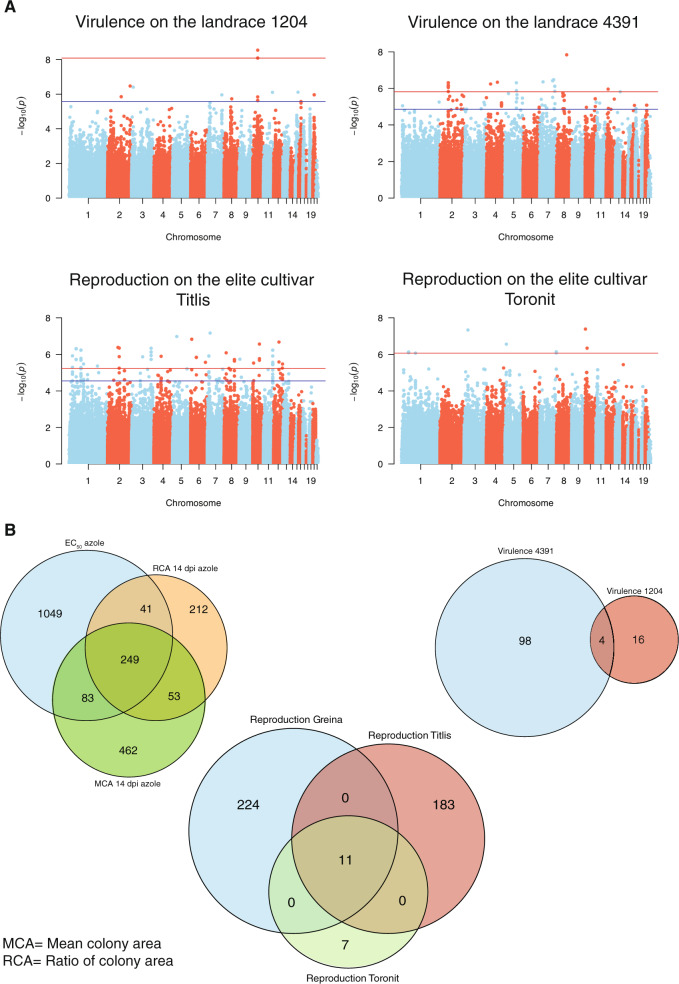
Genome-wide association mapping (GWAS) for 50 traits measured in various host and non-host environments. **A** Manhattan plots showing SNP marker association *p* values for four individual traits. The blue and red lines indicate the significant thresholds for false discovery rates (FDR) at 10 and 5%, respectively. **B** Venn diagrams showing the number of SNPs significantly associated with multiple traits (FDR 10%). Virulence (amount of necrotic lesion area) and reproduction (pycnidia density within the lesion area) were measured on 12 different wheat hosts.

To directly identify loci associated with phenotypic trait variation, we performed GWAS for each trait individually using a mixed linear model. Because the mapping population showed genetic heterogeneity due to population structure, we included the GRM as a random factor to control for *p* value inflation (see Methods for more details on the procedure). For the majority of the traits, including virulence, reproduction and fungicide resistance, we detected between 18 and 1422 significantly associated SNPs at a 10% false discovery rate (FDR) threshold. We identified no phenotype-genotype association at the very stringent Bonferroni threshold (α = 0.05). This suggests that most traits have a polygenic architecture with many loci of small effect. The large set of GWAS on individual traits allows to search for SNPs significantly associated with more than one trait. Such shared SNPs can point to a shared genetic control among traits or pleiotropic effects. We detected 249 SNPs shared among fungicide resistance traits including colony growth (i.e., mean colony area at 14 dpi at 22 °C and the ratio of colony area at 14 dpi in presence of fungicides; Fig. 3A, B**;** Supplementary Table S6). Importantly, we detected four SNPs associated with virulence on both the landraces 1204 and 4391, as well as 11 SNPs associated with reproduction on the elite cultivars Greina, Titlis and Toronit (Fig. 3B). To investigate the nature of the significantly associated SNPs among traits, we analyzed the genes in close physical proximity to each SNP. We used a cut-off of 1 kb between SNPs and genes to be considered. *Z. tritici* populations show (1) substantial linkage disequilibrium at this distance and (2) the average distance between genes in the genome is ~1 kb [30, 32]. Based on these criteria, we found a total of 781 genes in proximity to all the SNPs above the 10% FDR threshold (Supplementary Table S7). A total of 32.7% of the genes did not encode a conserved protein domain (PFAM). The remaining genes encoded a broad range of functions including transcription initiation domains, protein kinase domains, zinc and sugar transporters, as well as major facilitator superfamily (MFS). Hence, the identified functions serve among others to control various metabolic functions and regulatory processes.

We identified a SNP (*7_1897774*) significantly associated both with fungicide resistance (i.e., EC_50_ azole) and colony growth (i.e., mean colony area at 14 dpi in presence of azole), which overlaps with the coding sequence of a protein controlling virulence on a specific wheat cultivar (*Avr3D1*; [74]). It remains unknown how shared control of these traits is governed at the molecular level. We found no significantly associated SNP (10% FDR) for virulence and reproduction in physical proximity of genes encoding known virulence factors including *AvrStb6* [75], *Avr3D1* [74] and *Zt09_8_00609* [30]. To further investigate fungicide resistance trade-offs, we identified 27 SNPs significantly associated with colony growth (i.e., mean colony area) and 26 SNPs significantly associated with the ratio of colony area in presence of azole in close proximity to the *CYP51* gene encoding the target of azole fungicides [71]. Among the four significantly associated SNPs shared between virulence on the landraces 1204 and 4391, the SNP (*8_1221638*) overlapped with a gene encoding a papain-like cysteine peptidase superfamily (*Zt09_8_00399*). The most significant SNPs shared for reproduction on the elite cultivars Greina, Titlis and Toronit overlapped with a gene encoding an autophagy-related protein (*Zt09_1_00319*). For fungicide related traits, the most significant shared SNPs overlapped with genes encoding an effector candidate (*Zt09_2_00572)* and a prenyltransferase (squalene oxidase repeat protein family; *Zt09_7_00426*). How such gene functions could facilitate trait expression across different environments remains to be elucidated. We performed gene ontology enrichment analysis of all gene sets associated with individual traits or gene sets close to SNPs shared among different traits. We found one significantly enriched gene ontology for a single trait. The enrichment was for fungicide resistance (i.e., EC_50_ azole) and showed an over-representation of phosphorus-containing transfer functions (*p* value = 0.0097; Supplementary Table S8). The lack of single major effect loci for individual traits or loci shared among traits suggests that shared genetic control or potential pleiotropic effects are governed by a broad range of different functions contributing to trait variation.

### Trait correlations and complex genetic control across environments

**Fig. 4:**
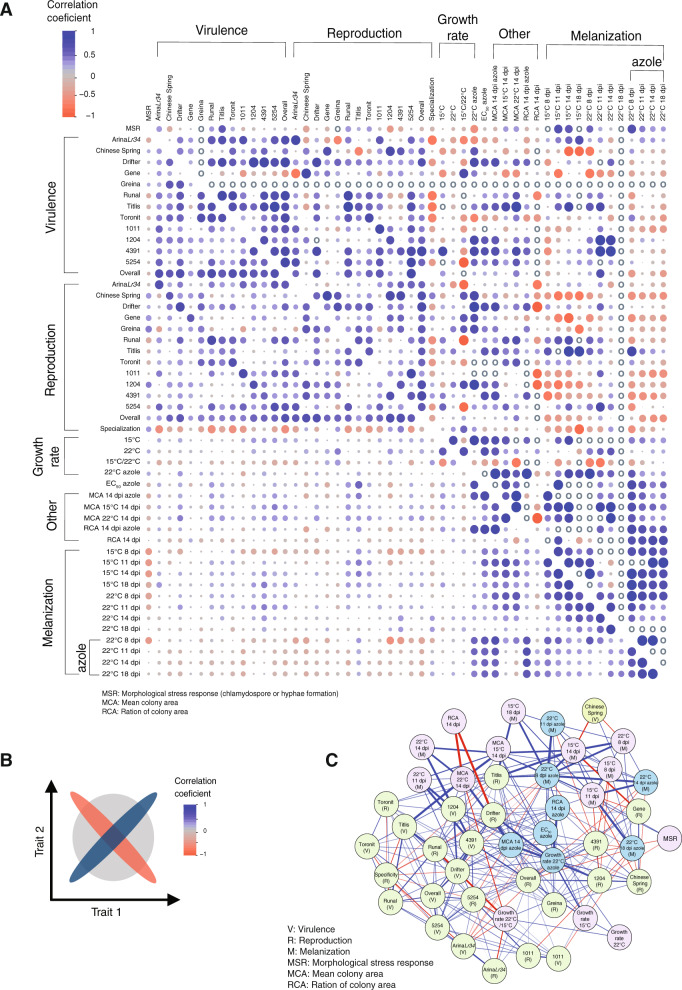
Correlations among 50 traits measured in various host and non-host environments. **A** Genetic correlation coefficients (upper diagonal) were estimated by following a bivariate genomic based restricted maximum likelihood (GREML) approach. Phenotypic correlation coefficients (lower diagonal) were estimated using standardized phenotypic values. Blue and red colors indicate positive and negative correlation coefficients, respectively. The circle size and color intensities are proportional to the correlation coefficient. The gray circles indicate “NA” as genetic correlation coefficients were out of bounds (i.e., −1 to 1) and also could not be estimated as the bivariate REML failed to converge. **B** A schematic representation of correlation between two traits. **C** Network of genetic correlation coefficients that were significantly different from zero at a 95% confidence interval. Pathogen virulence (amount of necrotic lesion area) and reproduction (pycnidia density within the lesion area) were measured on 12 different wheat hosts.

To expand our understanding how loci across the genome impact phenotypic trait variation, we analyzed correlation among traits both at the phenotypic and genetic level. Overall, both genetic correlation between traits (*r*_*g*_ = −0.98 to 0.98) and phenotypic correlation (*r*_*p*_ = −0.83 to 0.91, 0.99 ≤ *P* ≤ 3.01e^−45^) were highly variable (Supplementary Tables S9 and S10). Correlations among traits at the phenotypic level can be confounded by population structure. This is because trait variation among populations is potentially obscured by genome-wide genetic differentiation. Hence, we focused also on genetic correlations by taking genetic relatedness explicitly into account. For this, we used a genome-based restricted maximum likelihood approach incorporating genetic relatedness estimated from genome-wide SNPs and individual phenotypic trait values (Fig. 4A). The comparison among all phenotypic trait pairs revealed a wide spectrum of strongly negative and positive correlations. Both phenotypic and genetic correlations are mostly positive suggesting partially shared genetic control for the expression of virulence and reproduction traits across the 12 wheat cultivars. There are notable negative correlations in particular with host specialization and traits expressed on cultivar Gene. Overall, we found a significant genetic trade-off between specialization in reproduction and overall virulence (*r*_*g*_ = −0.67, SE = 0.21). Negative correlations may underlie conflicts in the expression of different pathogenicity traits possibly related to polymorphism at the level of the host immune system. The network representation of the genetic correlations reveals particularly strong negative correlations for ratio of colony area in presence of azole with colony growth and reproduction on the wheat cultivar Drifter (Fig. 4C). Colony growth traits show general strong positive correlation as expected but are also negatively correlated for pathogenicity on wheat cultivars such as Toronit, Arina*Lr34* and the landrace 5254 (Fig. 4B, C). The moderate (*r*_*g*_ = 0.34, SE = 0.29) to strong (*r*_*g*_ = 0.69, SE = 0.29) genetic correlation coefficients between morphological stress response (i.e., chlamydospore formation or hyphal growth) and colony melanization in multiple environments suggests a shared genetic control for these traits. Furthermore, fungicide resistance showed a negative genetic correlation with virulence on the cultivar Arina*Lr34* (*r*_*g*_ = −0.32, SE = 0.15) and Toronit (*r*_*g*_ = −0.38, SE = 0.30), reproduction on the landrace Chinese Spring (*r*_*g*_ = −0.26, SE = 0.16) and the degree of reproduction specialization (*r*_*g*_ = −0.19, SE = 0.18). Overall colony growth rates at 22 °C had mostly very low genetic (often negative) and phenotypic correlation coefficients with pathogenicity traits. This suggests that mutations favoring colony growth may not favor overall plant colonization. Overall, the observed genetic correlations suggest that pleiotropic effects across multiple genes could govern major phenotypic traits of the pathogen.

**Fig. 5:**
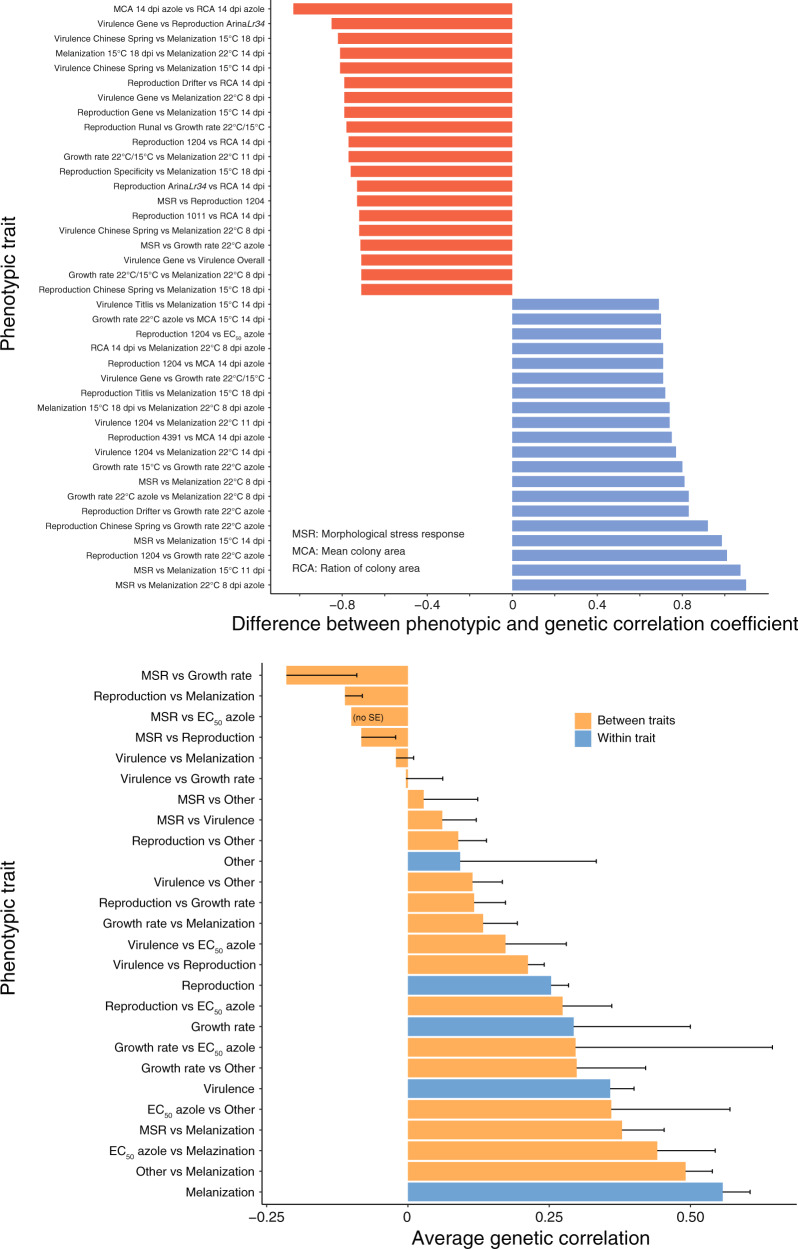
Discordance between genetic and phenotypic correlations. **A** Bar plot showing the difference between genetic and phenotypic correlation coefficients. A total of 20 trait pairs from each tail of the distribution (*n* = 1049 pairings) were selected for visualization. **B** Bar plot showing the average genetic correlation (relative degree of overlapping loci) within and between major trait categories. Pathogen virulence (amount of necrotic lesion area) and reproduction (pycnidia density within the lesion area) were measured on 12 diverse wheat hosts. Error bars indicate positive standard errors only. MSR vs EC_50_ has no standard error (single traits).

We quantified the difference between phenotypic and genetic correlations for each pair of traits. Focusing on the most extreme differences (Fig. 5A), we found a reduction of −1.03 in the genetic correlation for colony growth (i.e., mean colony area) and fungicide resistance (i.e., ratio of colony area in presence of fungicide at 14 dpi; *r*_*g*_ = −0.22, *r*_*p*_ = 0.81). We also found an excess of 1.1 in the genetic correlation for the morphological stress response and melanization at 22 °C at 8 dpi in presence of fungicide (*r*_*g*_ = 0.69, *r*_*p*_ = −0.41). Such disparity can occur when environmental effects (i.e., non-genetic additive effects) act in the opposite direction of genetic effects. Overall, we detected 20 pairs of traits with genetic correlation coefficients showing sign changes (e.g., positive to negative) compared to the corresponding phenotypic correlation coefficients. Given the discordance between phenotype and genetic correlations, we therefore used only genetic correlation coefficients for the subsequent analyses. The average genetic correlation for trait categories can be informative for the degree of shared genetic control of the traits (Fig. 5B). The average genetic correlation for virulence (0.35, SE = 0.04) was higher than for growth rate (0.29, SE = 0.2). This indicates that plant colonization exhibit stronger correlations among different hosts than colony growth outside of the host. The highest average genetic correlation was found for melanization (0.55, SE = 0.04) followed by melanization and associated traits for colony area (other; 0.49, SE = 0.04), fungicide resistance and melanization (0.44, SE = 0.1). Our findings show that the genetic architecture of melanization, fungicide resistance and colony growth (i.e., area) across environments is strongly overlapping.

**Fig. 6:**
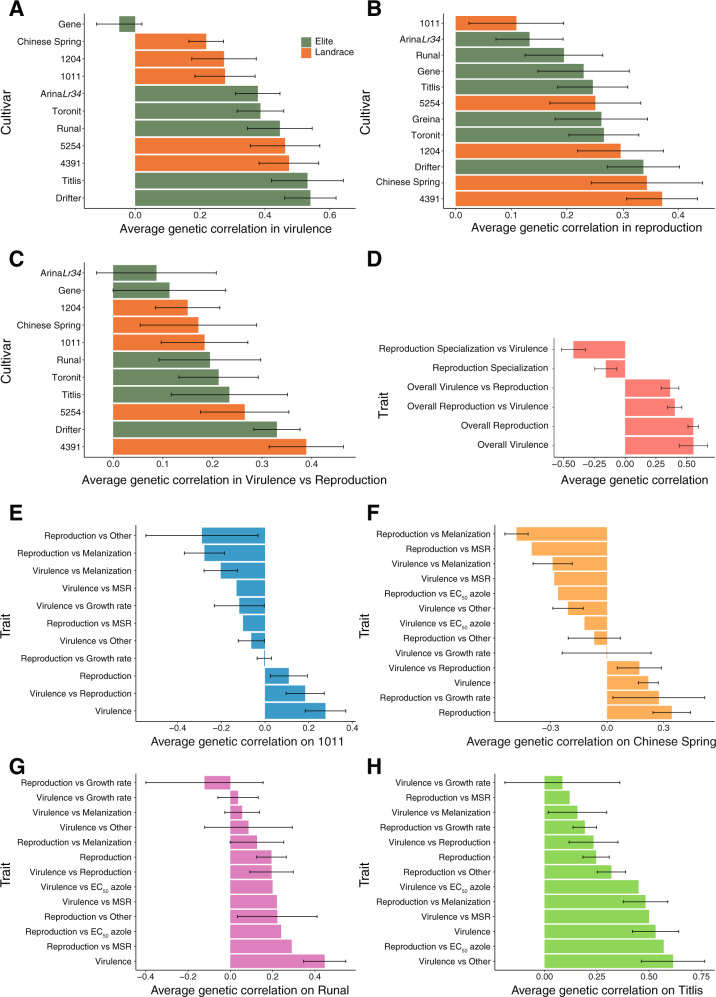
Average genetic correlation among host adaptation traits and environmental stress traits. Bar plots represent shared adaptive loci inferred from average genetic correlation coefficients for **A** pathogen virulence (amount of necrotic lesion area), **B** reproduction (pycnidia density within the lesion area), **C** between virulence and reproduction on each of the 12 wheat hosts, **D** overall virulence and reproduction combined over 12 hosts and host specialization. Host specialization is represented as the coefficient of variation of means across 12 hosts. **E**–**H** Bar plots showing the relative degree of interaction between the genetic control of host and non-host traits on each of the 12 hosts. Only four hosts are shown and the eight remaining hosts are shown in Supplementary Fig. 1. Melanization was expressed on a grayscale ranging from 0 (white) to 255 (black). Error bars indicate standard errors.

In plant-pathogen interactions, the genetic background of the host and pathogen often contributes to strong interactions at the genetic level (i.e., gene-for-gene interactions). Hence, we analyzed how strongly pathogenicity traits correlate between hosts (Fig. 6A–C). We found that the average genetic correlation for virulence and reproduction on the elite cultivar Gene and the landrace 1011 (−0.04, SE = 0.07; 0.10, SE = 0.08, respectively) and the average genetic correlation between virulence and reproduction on the elite cultivar Arina*Lr34* (0.08, SE = 0.12) were particularly low compared to the other cultivars. This indicates that the genetic control of pathogenicity traits on these hosts is the most differentiated compared to the other hosts. The negative average genetic correlation for reproduction specialization (−0.15, SE = 0.09) is in contrast to the positive overall average genetic correlation for virulence (0.55, SE = 0.11) and reproduction (0.55, SE = 0.04) (Fig. 6D). This suggests antagonistic pleiotropy in the genetic control of host specialization. We further investigated how genetic control for pathogenicity on different hosts overlaps with genetic control of colony growth, temperature sensitivity and fungicide resistance (Fig. 6E–H & Supplementary Fig. 1). We found a positive average genetic correlation for all the above traits on the elite cultivars Titlis and Runal (except the average genetic correlation between reproduction and growth rate). This suggests synergistic pleiotropy of pathogenicity and performance in absence of the host (i.e., stress response, thermal sensitivity and growth). Pathogenicity on the other hosts shows a wide range of positive and negative average genetic correlation between different traits. Hence, SNPs associated with increased virulence and reproduction on specific hosts can have antagonistic effects on other traits such as growth or melanization. This suggests that a complex mix of antagonistic and synergistic pleiotropy underlies the evolution of pathogenicity.

## Discussion

A mechanistic view of the adaptive landscape across the life cycle is of fundamental importance to understand pathogen evolution [76]. Yet, we lack fundamental insights into the underlying genetic architecture and how adaptation may be constrained. Here, we report the first study that maps a broad range of adaptive traits in a pathogen using a large-scale multi-trait GWAS. We show that the expression of most life-history traits is highly variable at the population level and has a largely polygenic basis. We found strong evidence of genetic trade-offs and facilitation among the life-history traits.

### Polygenic architecture of life-history trait expression

We show that a substantial proportion of the overall phenotypic variation is explained by common variants (i.e., SNPs with minor allele frequency > 0.05), consistent with polygenic trait architectures with many loci of small individual effect [65, 66, 77–79]. A polygenic architecture for major life history traits means that adaptation across the diverse environments on and off the host likely proceeds via subtle shifts in allele frequencies across many loci [80]. The polygenic nature of most traits was further supported by the identification of loci controlling traits measured in multiple host and non-host environments. We found that heritability was higher for pathogen reproduction than pathogen virulence (i.e., damage to the host). This may be explained by more differentiated host exploitation strategies for reproduction contributing to the maintenance of variation within pathogen populations [24]. The fact that both the host immune system and nutrient availability will have dominant effects on reproduction could also explain the high variability in reproduction. In contrast, a lack of variation among the isolates for causing damage (i.e., virulence) on a highly susceptible cultivar such as Greina can result in heritability of near zero. Higher heritability for pathogen reproduction on hosts means a potentially faster response to selection and ultimately host adaptation. However, selection on reproduction should also impact virulence and transmission through correlated responses [3, 24, 81, 82]. Genetic variation for traits expressed outside of the host such as fungicide resistance, thermal adaptation, and stress responses is thought to facilitate pathogen adaptation to abiotic stress. Maintenance of such variation is particularly relevant as pathogen populations face potential bottlenecks in-between the annual wheat growing seasons [33, 83]. A relevant consideration is whether the homogenization of agricultural landscapes impacts pathogen evolution [84]. Homogenization of the environment can increase directional selection pressure and erode heritable variation for life history traits. How *Z. tritici* retains such high levels of adaptive genetic variation along environmental gradients remains unclear. A key factor maintaining variation may be genetic trade-offs among life history traits constraining directional selection [85, 86].

### The adaptive landscape is shaped by extensive genome-wide pleiotropy

Adaptation to complex environments can be severely constrained by genetic trade-offs, but populations can also experience rapid adaptation through correlated responses to selection if genetic correlations are positive [4, 5, 87, 88]. We show that adaptation of *Z. tritici* is both constrained and facilitated by genetic correlations that vary according to the life-history traits and the environment. Unlike phenotypic correlations, genetic correlations estimated using mixed models are robust to confounding effects of population structure and directly reflect the genetic contributions to adaptive trait co-variation [89, 90]. Given the largely polygenic architecture of most life history traits, the observed genetic correlations are most likely caused by pleiotropic effects of numerous small effect loci. Alternatively, genetic correlations could be controlled by nearby loci in linkage disequilibrium. However, such genetic correlations should be transient and broken down rapidly by the high recombination rate of *Z. tritici* [33, 91, 92]. Overall, the magnitude of trade-offs and facilitation varied considerably among biotic and abiotic environments, and life cycle stages. Precise knowledge of genetic correlations can identify trade-offs and through this help to predict the evolutionary trajectories of pathogen populations.

A major finding on the evolution of pathogenicity is the positive correlation between virulence and reproduction. Such correlation could stem from convergent evolution selecting for similar synergistic mutations in shared resistant backgrounds of cultivated wheat worldwide [23, 93]. Moreover, a positive genetic correlation between virulence and reproduction could be attributed to the fact that this pathogen typically reproduces on damaged (i.e., necrotic) leaf tissue, hence mutations contributing to higher damage could facilitate reproduction at a later stage. It is important to note though that virulence and reproduction are highly variable traits across host resistance backgrounds because host factors have an important impact on the pathogen’s ability to cause damage and reproduce [41]. The considerable overlap in shared adaptive loci likely facilitates rapid adaptation to diverse host backgrounds. Adaptation to resistant hosts such as Gene or Arina*Lr34* or the landrace 1011 very likely imposed selection pressure on pathogen populations to evolve a distinct, host-specific arsenal of virulence factors. Consistent with theoretical predictions [94, 95], our study suggests that adaptation to novel hosts will be constrained due to strong antagonistic effects of host specialization for reproduction. Furthermore, adaptation to new abiotic environments is likely to be similarly constrained. Host or niche specialization is a driver of pathogen local adaptation, yet antagonistic effects can result in pathogen maladaptation when faced with nonoptimal environments [23]. In contrast to other pathosystems (e.g., [96, 97]), genetic control of *Z. tritici* growth outside of the host was largely independent and distinct from traits promoting host infection indicating that very few loci show pleiotropic effects. Nevertheless, on some host backgrounds, the ability to cause disease could be constrained by the pleiotropic effects of stress-related traits such as fungicide resistance or melanization. Genetic constraints governing growth in the absence of the host and pathogenicity could be a major impediment for pathogen populations to maximize fitness on hosts. This would be due to the fact that fitness outside the host is affected by correlated responses to selection on traits expressed on the host. The finding that *Z. tritici* populations carry variants with negative pleiotropic effects for infection despite apparent costs is intriguing. Variants underlying strong stress-related trait expression variation should be highly selectable and are likely underpinning survival under harsh climate as shown by strong synergistic pleiotropic effects. Therefore, maladaptive trait expression on the host could well be maintained in pathogen populations if the underlying variants are favored under harsh climatic conditions [98–100].

## Conclusion

Our study reveals remarkable complexity in pleiotropic effects across the life cycle in a major eukaryotic plant pathogen. Furthermore, adaptation to host and non-host environments has a largely polygenic basis. Our study demonstrates the power of conducting a large-scale GWAS across a wide range of environmental conditions. We show that trade-offs are not ubiquitous, but rather facilitation (i.e., synergistic pleiotropy) plays an important role in determining the evolutionary potential of pathogen populations across environments. By leveraging information on facilitation and trade-offs, the response of pathogens to changing environments becomes more predictable. Furthermore, knowledge on trade-offs can be exploited in innovative disease management models that exploit evolutionary weaknesses of pathogens. For example, trade-offs between pathogen virulence on specific cultivars and fungicide resistance could be exploited by planting specific wheat genotypes in areas where fungicide resistance emergence becomes a risk. A clear understanding of facilitation and correlated responses to selection can also be applied to prevent catastrophic breakdowns in resistance. The antagonisms among life-history traits on different hosts can inform crop resistance management by spatially or temporally diversifying agricultural fields with different hosts according to the prevalent environmental conditions. By creating complex but specifically designed host environments, pathogen populations could be constrained in their ability to cause serious host damage. Retracing specific biochemical and developmental pathways underpinning pleiotropic effects is greatly facilitated by the multi-trait genome-wide mapping approach and will provide a deeper insight into principles governing pathogen adaptation in heterogeneous environments.

## Supplementary information

Supplementary Information

Supplementary Tables

## Data Availability

All genome sequences are available from the NCBI Sequence Read Archive (BioProject accessions PRJNA327615, PRJNA596434, and PRJNA178194).

## References

[1] Laine AL, Barrès B (2013). Epidemiological and evolutionary consequences of life‐history trade‐offs in pathogens. Plant Pathol.

[2] Lannou C. Variation and selection of quantitative traits in plant pathogens. Ann Rev Phytopathol. 2012;50:319–38. 10.1146/annurev-phyto-081211-173031 22702351

[3] Anderson RM, May RM (1982). Coevolution of hosts and parasites. Parasitology.

[4] Roff DA (2000). Trade-offs between growth and reproduction: an analysis of the quantitative genetic evidence. J Evolut Biol.

[5] Stearns SC (1989). Trade-offs in life-history evolution. Funct Ecol.

[6] Hughes KA, Leips J (2017). Pleiotropy, constraint, and modularity in the evolution of life histories: insights from genomic analyses. Ann N Y Acad Sci.

[7] Trivedi P, Wang N (2014). Host immune responses accelerate pathogen evolution. ISME J.

[8] Rodríguez-Verdugo A, Carrillo-Cisneros D, González-González A, Gaut BS, Bennett AF (2014). Different tradeoffs result from alternate genetic adaptations to a common environment. Proc Natl Acad Sci USA.

[9] Hill JA, O’Meara TR, Cowen LE (2015). Fitness trade-offs associated with the evolution of resistance to antifungal drug combinations. Cell Rep.

[10] Jackson JC, Higgins LA, Lin X. Conidiation color mutants of *Aspergillus fumigatus* are highly pathogenic to the heterologous insect host *Galleria mellonella*. PLoS ONE. 2009; p. e4224. 10.1371/journal.pone.0004224 PMC2625396 19156203

[11] Wagner GP, Zhang J (2011). The pleiotropic structure of the genotype–phenotype map: the evolvability of complex organisms. Nat Rev Genet.

[12] Wang Z, Liao BY, Zhang J (2010). Genomic patterns of pleiotropy and the evolution of complexity. Proc Natl Acad Sci USA.

[13] Roff DA (2007). Contributions of genomics to life-history theory. Nat Rev Genet.

[14] Saltz JB, Hessel FC, Kelly MW (2017). Trait correlations in the genomics era. Trends Ecol evolution.

[15] Durmaz E, Rajpurohit S, Betancourt N, Fabian DK, Kapun M, Schmidt P (2019). A clinal polymorphism in the insulin signaling transcription factor foxo contributes to life‐history adaptation in Drosophila. Evolution.

[16] Fletcher RS, Mullen JL, Heiliger A, McKay JK (2015). QTL analysis of root morphology, flowering time, and yield reveals trade-offs in response to drought in *Brassica napus*. J Exp Bot.

[17] Lendenmann MH, Croll D, McDonald BA (2015). QTL mapping of fungicide sensitivity reveals novel genes and pleiotropy with melanization in the pathogen *Zymoseptoria tritici*. Fungal Genet Biol.

[18] Scarcelli N, Cheverud JM, Schaal BA, Kover PX (2007). Antagonistic pleiotropic effects reduce the potential adaptive value of the FRIGIDA locus. Proc Natl Acad Sci USA.

[19] Chen P, Zhang J (2020). Antagonistic pleiotropy conceals molecular adaptations in changing environments. Nat Ecol Evol.

[20] Hill WG, Zhang XS (2012). On the pleiotropic structure of the genotype–phenotype map and the evolvability of complex organisms. Genetics.

[21] Fones H, Gurr S (2015). The impact of Septoria tritici Blotch disease on wheat: An EU perspective. Fungal Genet Biol.

[22] Torriani SF, Melichar JP, Mills C, Pain N, Sierotzki H, Courbot M (2015). *Zymoseptoria tritici*: a major threat to wheat production, integrated approaches to control. Fungal Genet Biol.

[23] Croll D, McDonald BA (2017). The genetic basis of local adaptation for pathogenic fungi in agricultural ecosystems. Mol Ecol.

[24] Dutta A, Croll D, McDonald BA, Barrett LG (2020). Maintenance of variation in virulence and reproduction in populations of an agricultural plant pathogen. Evol Appl.

[25] Zhan J, Stefanato FL, McDonald BA (2006). Selection for increased cyproconazole tolerance in *Mycosphaerella graminicola* through local adaptation and in response to host resistance. Mol Plant Pathol.

[26] Zhan J, McDonald BA (2011). Thermal adaptation in the fungal pathogen *Mycosphaerella graminicola*. Mol Ecol.

[27] Krishnan P, Meile L, Plissonneau C, Ma X, Hartmann FE, Croll D (2018). Transposable element insertions shape gene regulation and melanin production in a fungal pathogen of wheat. BMC Biol.

[28] Francisco CS, Ma X, Zwyssig MM, McDonald BA, Palma-Guerrero J (2019). Morphological changes in response to environmental stresses in the fungal plant pathogen *Zymoseptoria tritici*. Sci Rep.

[29] Sánchez‐Vallet A, Hartmann FE, Marcel TC, Croll D (2018). Nature’s genetic screens: using genome‐wide association studies for effector discovery. Mol plant Pathol.

[30] Hartmann FE, Sánchez-Vallet A, McDonald BA, Croll D (2017). A fungal wheat pathogen evolved host specialization by extensive chromosomal rearrangements. ISME J.

[31] Badet T, Oggenfuss U, Abraham L, McDonald BA, Croll D (2020). A 19-isolate reference-quality global pangenome for the fungal wheat pathogen *Zymoseptoria tritici*. BMC Biol.

[32] Goodwin SB, M’Barek SB, Dhillon B, Wittenberg AH, Crane CF, Hane JK (2011). Finished genome of the fungal wheat pathogen *Mycosphaerella graminicola* reveals dispensome structure, chromosome plasticity, and stealth pathogenesis. PLoS Genet.

[33] Zhan J, Linde CC, Jürgens T, Merz U, Steinebrunner F, McDonald BA (2005). Variation for neutral markers is correlated with variation for quantitative traits in the plant pathogenic fungus *Mycosphaerella graminicola*. Mol Ecol.

[34] Linde CC, Zhan J, McDonald BA (2002). Population structure of *Mycosphaerella graminicola*: from lesions to continents. Phytopathology.

[35] Bolger AM, Lohse M, Usadel B (2014). Trimmomatic: a flexible trimmer for Illumina sequence data. Bioinformatics.

[36] Langmead B, Trapnell C, Pop M, Salzberg SL (2009). Ultrafast and memory-efficient alignment of short DNA sequences to the human genome. Genome Biol.

[37] McKenna A, Hanna M, Banks E, Sivachenko A, Cibulskis K, Kernytsky A (2010). The genome analysis toolkit: a MapReduce framework for analyzing next-generation DNA sequencing data. Genome Res.

[38] Hartmann FE, McDonald BA, Croll D (2018). Genome-wide evidence for divergent selection between populations of a major agricultural pathogen. Molecular Ecology.

[39] McIntosh RA (1992). Close genetic linkage of genes conferring adult‐plant resistance to leaf rust and stripe rust in wheat. Plant Pathol.

[40] Spielmeyer W, McIntosh RA, Kolmer J, Lagudah ES (2005). Powdery mildew resistance and *Lr34/Yr18* genes for durable resistance to leaf and stripe rust cosegregate at a locus on the short arm of chromosome 7D of wheat. Theor Appl Genet.

[41] Karisto P, Hund A, Yu K, Anderegg J, Walter A, Mascher F (2018). Ranking quantitative resistance to Septoria tritici blotch in elite wheat cultivars using automated image analysis. Phytopathology.

[42] Francisco, CS, McDonald, BA, Palma-Guerrero, J. Regulatory mechanisms of morphological transitions in response to temperature stress in a fungal pathogen. 2020. *In preparation.*

[43] Lendenmann MH, Croll D, Palma-Guerrero J, Stewart EL, McDonald BA (2016). QTL mapping of temperature sensitivity reveals candidate genes for thermal adaptation and growth morphology in the plant pathogenic fungus *Zymoseptoria tritici*. Heredity.

[44] Lendenmann MH, Croll D, Stewart EL, McDonald BA (2014). Quantitative trait locus mapping of melanization in the plant pathogenic fungus *Zymoseptoria tritici*. *G3: Genes, Genomes*. Genetics.

[45] Mohd‐Assaad N, McDonald BA, Croll D (2016). Multilocus resistance evolution to azole fungicides in fungal plant pathogen populations. Mol Ecol.

[46] Ritz C, Baty F, Streibig JC, Gerhard D (2015). Dose-response analysis using R. PloS One.

[47] R Core Team. (2014). R: a language and environment for statistical computing.

[48] Döring TF, Reckling M (2018). Detecting global trends of cereal yield stability by adjusting the coefficient of variation. Eur J Agron.

[49] Gu Z, Eils R, Schlesner M (2016). Complex heatmaps reveal patterns and correlations in multidimensional genomic data. Bioinformatics.

[50] Bradbury PJ, Zhang Z, Kroon DE, Casstevens TM, Ramdoss Y, Buckler ES (2007). TASSEL: software for association mapping of complex traits in diverse samples. Bioinformatics.

[51] Yang J, Lee SH, Goddard ME, Visscher PM (2011). GCTA: a tool for genome-wide complex trait analysis. Am J Hum Genet.

[52] Schwarz G (1978). Estimating the dimension of a model. Ann Stat.

[53] Tang, Y, Liu, X, Wang, J, Li, M, Wang, Q, Tian, F, et. al. GAPIT version 2: an enhanced integrated tool for genomic association and prediction. Plant Genome. 2016;9:1–9. 10.3835/plantgenome2015.11.0120 27898829

[54] Yu J, Pressoir G, Briggs WH, Bi IV, Yamasaki M, Doebley JF (2006). A unified mixed-model method for association mapping that accounts for multiple levels of relatedness. Nat Genet.

[55] Zhang Z, Ersoz E, Lai CQ, Todhunter RJ, Tiwari HK, Gore MA (2010). Mixed linear model approach adapted for genome-wide association studies. Nat Genet.

[56] Storey JD, Tibshirani R (2003). Statistical significance for genomewide studies. Proc Natl Acad Sci USA.

[57] Benjamini Y, Hochberg Y (1995). Controlling the false discovery rate: a practical and powerful approach to multiple testing. J R Stat Soc: Ser B.

[58] Grandaubert J, Bhattacharyya A, Stukenbrock EH (2015). RNA-seq-based gene annotation and comparative genomics of four fungal grass pathogens in the genus Zymoseptoria identify novel orphan genes and species-specific invasions of transposable elements. G3: Genes Genomes Genet.

[59] Quinlan AR, Hall IM (2010). BEDTools: a flexible suite of utilities for comparing genomic features. Bioinformatics.

[60] Jones P, Binns D, Chang HY, Fraser M, Li W, McAnulla C (2014). InterProScan 5: genome-scale protein function classification. Bioinformatics.

[61] Nielsen H. Predicting secretory proteins with SignalP. In Protein function prediction (pp. 59-73). New York, NY: Humana Press; 2107.

[62] Käll L, Krogh A, Sonnhammer EL (2007). Advantages of combined transmembrane topology and signal peptide prediction—the Phobius web server. Nucleic acids Res.

[63] Krogh A, Larsson B, Von Heijne G, Sonnhammer EL (2001). Predicting transmembrane protein topology with a hidden Markov model: application to complete genomes. J Mol Biol.

[64] Falcon S, Gentleman R (2007). Using GOstats to test gene lists for GO term association. Bioinformatics.

[65] Yang J, Benyamin B, McEvoy BP, Gordon S, Henders AK, Nyholt DR (2010). Common SNPs explain a large proportion of the heritability for human height. Nat Genet.

[66] Yang J, Zeng J, Goddard ME, Wray NR, Visscher PM (2017). Concepts, estimation and interpretation of SNP-based heritability. Nat Genet.

[67] Lee SH, Yang J, Goddard ME, Visscher PM, Wray NR (2012). Estimation of pleiotropy between complex diseases using single-nucleotide polymorphism-derived genomic relationships and restricted maximum likelihood. Bioinformatics.

[68] Epskamp S, Cramer AO, Waldorp LJ, Schmittmann VD, Borsboom D (2012). qgraph: Network visualizations of relationships in psychometric data. J Stat Softw.

[69] Kornbrot D. Point Biserial Correlation. Wiley StatsRef: Statistics Reference Online: New York, Wiley, 2014.

[70] Rizopoulos D (2006). ltm: An R package for latent variable modeling and item response theory analyses. J Stat Softw.

[71] Cools HJ, Fraaije BA (2012). Update on mechanisms of azole resistance in Mycosphaerella graminicola and implications for future control. Pest Manag Sci.

[72] Hartmann FE, Vonlanthen T, Singh NK, McDonald M, Milgate A, Croll D. The complex genomic basis of rapid convergent adaptation to pesticides across continents in a fungal plant pathogen. Molecular Ecology. 2020;00:1–16. 10.1111/mec.15737 33211369

[73] Pereira D, McDonald BA, Croll D. The genetic architecture of emerging fungicide resistance in populations of a global wheat pathogen. Genome Biol. Evol. 2020;12:2231–44. 10.1093/gbe/evaa203 PMC7846115 32986802

[74] Meile L, Croll D, Brunner PC, Plissonneau C, Hartmann FE, McDonald BA (2018). A fungal avirulence factor encoded in a highly plastic genomic region triggers partial resistance to septoria tritici blotch. N. Phytologist.

[75] Zhong Z, Marcel TC, Hartmann FE, Ma X, Plissonneau C, Zala M (2017). A small secreted protein in Zymoseptoria tritici is responsible for avirulence on wheat cultivars carrying the Stb6 resistance gene. N. Phytologist.

[76] Hartmann FE, Rodríguez de la Vega RC, Carpentier F, Gladieux P, Cornille A, Hood ME (2019). Understanding adaptation, coevolution, host specialization, and mating system in castrating anther-smut fungi by combining population and comparative genomics. Annu Rev Phytopathol.

[77] Korte A, Farlow A (2013). The advantages and limitations of trait analysis with GWAS: a review. Plant methods.

[78] Slate J (2013). From Beavis to beak color: a simulation study to examine how much QTL mapping can reveal about the genetic architecture of quantitative traits. Evolution.

[79] Wood AR, Esko T, Yang J, Vedantam S, Pers TH, Gustafsson S (2014). Defining the role of common variation in the genomic and biological architecture of adult human height. Nat Genet.

[80] Höllinger I, Pennings PS, Hermisson J (2019). Polygenic adaptation: from sweeps to subtle frequency shifts. PLoS Genet.

[81] Leggett HC, Buckling A, Long GH, Boots M (2013). Generalism and the evolution of parasite virulence. Trends Ecol Evol.

[82] Pettay JE, Kruuk LE, Jokela J, Lummaa V (2005). Heritability and genetic constraints of life-history trait evolution in preindustrial humans. Proc Natl Acad Sci USA.

[83] McDonald BA, Linde C (2002). Pathogen population genetics, evolutionary potential, and durable resistance. Annu Rev Phytopathol.

[84] McDonald BA, Stukenbrock EH (2016). Rapid emergence of pathogens in agro-ecosystems: global threats to agricultural sustainability and food security. Philos Trans R Soc B: Biol Sci.

[85] Reznick D (1992). Measuring reproductive costs: response to Partridge. Trends Ecol Evol.

[86] Roff DA (1992). The evolution of life histories: theory and analysis.

[87] McGee LW, Sackman AM, Morrison AJ, Pierce J, Anisman J, Rokyta DR. Synergistic pleiotropy overrides the costs of complexity in viral adaptation. Genetics. 2016;202:285–95. 10.1534/genetics.115.181628 PMC4701092 26564159

[88] Sgrò CM, Hoffmann AA (2004). Genetic correlations, tradeoffs and environmental variation. Heredity.

[89] Bulik-Sullivan B, Finucane HK, Anttila V, Gusev A, Day FR, Loh PR (2015). An atlas of genetic correlations across human diseases and traits. Nat Genet.

[90] van Rheenen W, Peyrot WJ, Schork AJ, Lee SH, Wray NR (2019). Genetic correlations of polygenic disease traits: from theory to practice. Nat Rev Genet.

[91] Zhan J, Pettway RE, McDonald BA (2003). The global genetic structure of the wheat pathogen *Mycosphaerella graminicola* is characterized by high nuclear diversity, low mitochondrial diversity, regular recombination, and gene flow. Fungal Genet Biol.

[92] Croll D, Lendenmann MH, Stewart E, McDonald BA (2015). The impact of recombination hotspots on genome evolution of a fungal plant pathogen. Genetics.

[93] Brown JK, Chartrain L, Lasserre-Zuber P, Saintenac C (2015). Genetics of resistance to *Zymoseptoria tritici* and applications to wheat breeding. Fungal Genet Biol.

[94] Kassen R (2002). The experimental evolution of specialists, generalists, and the maintenance of diversity. J Evolut Biol.

[95] Legros M, Koella JC (2010). Experimental evolution of specialization by a microsporidian parasite. BMC Evolut Biol.

[96] Thrall PH, Barrett LG, Burdon JJ, Alexander HM (2005). Variation in pathogen aggressiveness within a metapopulation of the *Cakile maritima–Alternaria brassicicola* host–pathogen association. Plant Pathol.

[97] Lee DH, Roux J, Wingfield BD, Wingfield MJ (2015). Variation in growth rates and aggressiveness of naturally occurring self‐fertile and self‐sterile isolates of the wilt pathogen *Ceratocystis albifundus*. Plant Pathol.

[98] Foster KR, Shaulsky G, Strassmann JE, Queller DC, Thompson CR (2004). Pleiotropy as a mechanism to stabilize cooperation. Nature.

[99] Frénoy A, Taddei F, Misevic D (2013). Genetic architecture promotes the evolution and maintenance of cooperation. PLoS Comput Biol.

[100] Wright S. Evolution and the genetics of populations, volume 1: genetic and biometric foundations (Vol. 1). University of Chicago press, Chicago; 1984.

