# Supplementary material for: Mapping the adaptive landscape of a major agricultural pathogen reveals evolutionary constraints across heterogeneous environments
Source: ISME J. 2021 Jan 15;15(5):1402–19. doi: 10.1038/s41396-020-00859-w (PMC8115182; doi:10.1038/s41396-020-00859-w)
Supplement: Supplementary file 1 — Supplementary Information [file 41396_2020_859_MOESM1_ESM.pdf]

Supplementary information

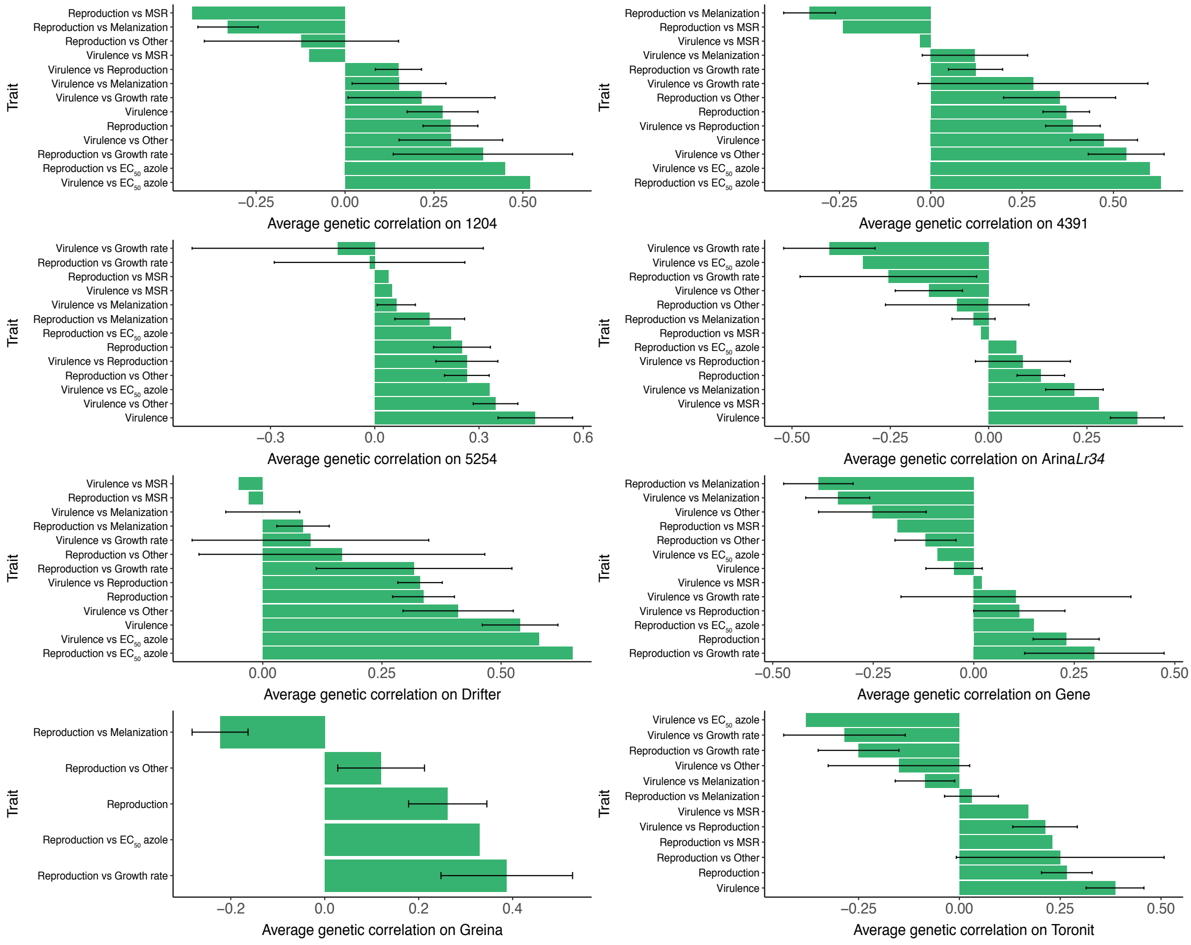

Supplementary Figure S1. Bar plots of average genetic correlations showing the interactions of genetic control between host and non-host traits on eight hosts. Pathogen virulence (amount of necrotic lesion area) and reproduction (pycnidia density within the lesion area) were measured on 12 diverse wheat hosts. Melanization was expressed on a grayscale ranging from 0 (white) to 255 (black). Error bars indicate standard errors.

Supplementary Tables

(see separate file "Supplementary_Tables.xlsx")

Supplementary Table S1. Description of 145 *Zymoseptoria tritici* isolates with corresponding sampling location, year and NCBI Sequence Read Archive accession number for the whole genome sequence data used in this study.

Supplementary Table S2. Raw phenotypic data for virulence (measured as the amount of necrotic lesion area) and reproduction (pycnidia density within lesion area) on 12 wheat cultivars from 145 *Zymoseptoria tritici* isolates.

Supplementary Table S3. Raw phenotypic data for mean colony area per plate and mean grey value per plate measured in different temperatures and in presence/absence of fungicide from 130 *Zymoseptoria tritici* isolates. "NA" indicates that no data were obtained due to no colony growth or contamination.

Supplementary Table S4. Number of principal components (PCs) included in the GWAS, the Bayesian Information Criteria (BIC) value and the log-likelihood values.

Supplementary Table S5. Least-square mean values of 50 traits based on raw phenotypic data from 145 Zymoseptoria tritici isolates.

Supplementary Table S6. Summary statistics of genome-wide SNPs crossing the false discovery rate (FDR) of 10% for specific traits. SNPs are ordered according to the smallest *P-value*.

Supplementary Table S7. Gene ontology (GO) term enrichment summary for fungicide resistance (EC50 azole) in *Zymoseptoria tritici*.

Supplementary Table S8. Estimates of genetic (upper diagonal) and phenotypic (lower diagonal) correlation co-efficients among 50 traits in various host and non-host environments. Genetic correlations were estimated using a bivariate genomic based restricted maximum likelihood (GREML) approach. Standard error associated with each genetic correlation coefficient is given inside the parenthesis. The "NA" indicates that genetic correlation coefficients were out of bounds (*i.e.* -1 to 1) and also could not be estimated as the bivariate REML failed to converge. Phenotypic correlations were estimated using standardized phenotypic values.

Supplementary Table S9. List of genes found in close proximity to all the SNPs above the false discovery rate (FDR) of 10%.

Supplementary Table S10. *P*-values for phenotypic correlation among 49 traits (except morphological stress response) adjusted for multiple testing following Benjamini-Hochberg method at α=0.05.
